# Establishment and evaluation of a rat model of extracorporeal membrane oxygenation (ECMO) thrombosis using a 3D-printed mock-oxygenator

**DOI:** 10.1186/s12967-021-02847-w

**Published:** 2021-04-28

**Authors:** Nao Umei, Angela Lai, Jennifer Miller, Suji Shin, Kalliope Roberts, Saif AI Qatarneh, Shingo Ichiba, Atsuhiro Sakamoto, Keith E. Cook

**Affiliations:** 1 Department of Anesthesiology and Pain Medicine, Graduate School of Medicine, Nippon Medical School, 1-1-5, Sendagi, Bunkyo-ku, Tokyo, 113-8603 Japan; 2 Department of Anesthesiology, Nippon Medical School, 1-1-5, Sendagi, Bunkyo-ku, Tokyo, 113-8603 Japan; 3 Department of Surgical Intensive Care Medicine, Nippon Medical School, 1-1-5, Sendagi, Bunkyo-ku, Tokyo, 113-8603 Japan; 4 Department of Biomedical Engineering, Carnegie Mellon University, 5000 Forbes Avenue, Pittsburgh, PA 15213 USA

**Keywords:** Extracorporeal membrane oxygenation, Rats, Three-dimensional printing, Thrombosis, Heparin, Anticoagulants

## Abstract

**Background:**

Extracorporeal membrane oxygenation (ECMO) research using large animals requires a significant amount of resources, slowing down the development of new means of ECMO anticoagulation. Therefore, this study developed and evaluated a new rat ECMO model using a 3D-printed mock-oxygenator.

**Methods:**

The circuit consisted of tubing, a 3D-printed mock-oxygenator, and a roller pump. The mock-oxygenator was designed to simulate the geometry and blood flow patterns of the fiber bundle in full-scale oxygenators but with a low (2.5 mL) priming volume. Rats were placed on arteriovenous ECMO at a 1.9 mL/min flow rate at two different heparin doses (n = 3 each): low (15 IU/kg/h for eight hours) versus high (50 IU/kg/h for one hour followed by 25 IU/kg/h for seven hours). The experiment continued for eight hours or until the mock-oxygenator failed. The mock-oxygenator was considered to have failed when its blood flow resistance reached three times its baseline resistance.

**Results:**

During ECMO, rats maintained near-normal mean arterial pressure and arterial blood gases with minimal hemodilution. The mock-oxygenator thrombus weight was significantly different (p < 0.05) between the low (0.02 ± 0.006 g) and high (0.003 ± 0.001 g) heparin delivery groups, and blood flow resistance was also larger in the low anticoagulation group.

**Conclusions:**

This model is a simple, inexpensive system for investigating new anticoagulation agents for ECMO and provides low and high levels of anticoagulation that can serve as control groups for future studies.

## Background

Extracorporeal membrane oxygenation (ECMO) is an essential therapy for supporting patients with severe cardiac and pulmonary failure refractory to conventional treatment [1].

Despite advancements in ECMO technology over the last decade, currently available commercial oxygenators last for only a few weeks due to thrombus formation [2–5]. While anticoagulation can be used to slow thrombus formation, its use must be significantly limited to avoid bleeding complications [1, 4, 5]. Therefore, there remains an urgent need for a simple animal ECMO model with which further research can be conducted to elucidate the role of fiber bundle design and various experimental anticoagulants to reduce thrombus formation.

Numerous animal models have been developed to study thrombus formation in oxygenators. Most of these are large animal models, such as goats, sheep, pigs, or cows [6–9]. The advantages of a larger animal are simpler surgical procedures, the use of commercially available oxygenators, and a far lower risk of oversampling blood and risking hypotension or hemodilution. However, these experiments require large, experienced laboratory staff, greater use of disposable resources, and a full-scale operating environment. As a result, these experiments are both expensive and challenging. The rabbit model reduces the cost and complexity of study but remains expensive compared to rodent research [8, 10].

For this reason, this study develops a highly miniaturized rat arteriovenous (A-V) ECMO model as a means to examine various methods of reducing thrombus formation in oxygenators. A rat ECMO model would enable a single person to perform experiments and require fewer resources, leading to a reduced cost. Furthermore, this allows the use of an animal that is lower on the phylogenetic scale and thus more ethically acceptable while offering a greater number of specialized genetic knockout and general disease models and hematologic assays. However, the rat model has limitations for investigating ECMO coagulopathy because of hemodilution caused by a large ECMO circuit priming volume.

Therefore, a small microfluidic device simulating an oxygenator was constructed for ECMO coagulation studies. The device in this study was constructed using 3D-printed, solid urethane acrylate. The simulated fiber bundle utilized solid, 380 µm diameter rods with a packing density of 50%, but this could be easily altered to investigate designs with different diameters and packing densities. Thus, the device has blood flow patterns and surface area to blood volume ratio within the simulated fiber bundle that are similar to those of commercial oxygenators but does not transfer gas. As such, this device is termed a “mock-oxygenator.” This device is inexpensive and can be built rapidly, has a priming volume of only 0.3 mL, but still possesses the blood flow patterns and surface area to blood volume ratio (0.005 m^2^/mL) that have a significant effect on thrombus formation in oxygenators. 3D-printed models of oxygenator components have previously been used to evaluate the blood flow resistance in fiber bundles and have only recently emerged as a means to evaluate thrombus formation [11, 12]. This study thus sought to evaluate the highly miniaturized A-V rat ECMO model consisting of a 3D-printed mock-oxygenator by comparing the thrombus formation and rat physiology at two heparin doses.

## Methods

### Animal care

Male Sprague–Dawley rats (Taconic Biosciences, Germantown, NY) received humane care in compliance with the Guide for the Care and Use of Laboratory Animals [13].

### Anesthesia and ventilation

Anesthesia was initiated with an intraperitoneal injection of ketamine (70 mg/kg) and xylazine (7.7 mg/kg). A small rectangular incision (0.5 × 1 cm^2^) was then made in the middle of the tail to expose the lateral tail vein [14]. A 24-gauge catheter (Insyte™ 0.7 × 19 mm, BD, Franklin Lakes, NJ) was inserted into the vein to maintain anesthesia throughout the experiment with a continuous intravascular injection of ketamine (1 mg/kg/min) and xylazine (0.03 mg/kg/min). Buprenorphine (0.01 mg/kg) was administered once subcutaneously for pain relief. Rats were intubated via tracheotomy with a 16-gauge catheter (Insyte™ 1.7 × 45 mm, BD) for mechanical ventilation (Small Animal Ventilator, Model 683, Harvard Apparatus, Holliston, MA) with a tidal volume of 7 mL/kg and rate of 65 breaths per minute using pure oxygen. The respiratory rate was adjusted to keep the partial pressure of arterial carbon dioxide (PaCO_2_) at 35–45 mm Hg.

### Cannulation

All rats were placed on pumped A-V ECMO. While this is not a typical mode of clinical ECMO attachment, it has advantages for surface coagulation testing in small animals. Blood drainage is easier to maintain from the higher blood pressure arteries, and including a pump allows these flows to be independent from the arterial pressure and thus carefully controlled in each rat. This system allows controllable, uninterrupted blood flow for evaluating circuit coagulation.

**Fig. 1:**
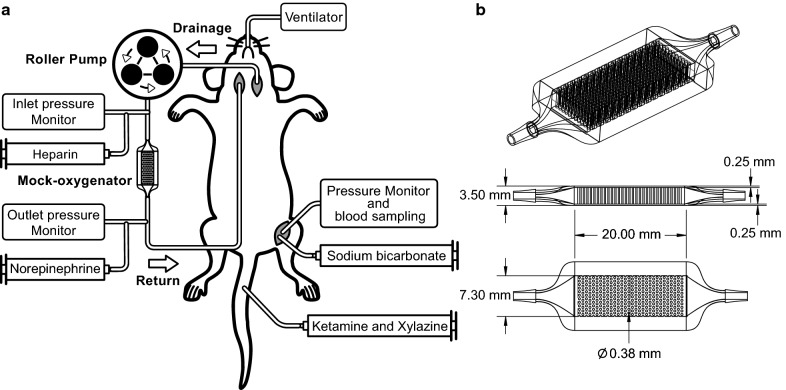
A rat model of extracorporeal membrane oxygenation thrombosis using a 3D-printed mock-oxygenator. Rat ECMO was established by cannulating the left carotid artery for access and the right jugular vein for return. The circuit consisted of a 1/16 inch ID tube, pump, and a 3D-printed mock-oxygenator. Pressure was monitored via T-connectors at the inlet and outlet of the simulated oxygenator. Heparin was administered through the inlet pressure line, and norepinephrine was administered through the outlet pressure line. Anesthetics were administered via the tail vein and sodium bicarbonate via the femoral vein. The femoral artery was used to monitor systemic arterial pressure and collect blood samples (**a**). The mock-oxygenator was designed with a 7.3 mm wide, 3 mm high, and 20 mm long flow chamber (**b**)

When sufficient anesthesia was achieved, the animals were positioned supine on a warming blanket. The core body temperature was monitored with a rectal probe and maintained at 37.5–38.5 °C by using a circulating warm water blanket (Homeothermic Blanket Systems with Flexible Probe, Harvard Apparatus) throughout the experiment. The carotid artery was cannulated with a 24-gauge drainage catheter (Insyte™ 0.7 × 19 mm, BD), which was advanced into the aortic arch and connected to the ECMO circuit inlet (see Fig. 1a below) [15]. The right external jugular vein was cannulated with a 20-gauge infusion catheter (Insyte™ 1.1 × 30 mm, BD), which was advanced to the bifurcation at the right external jugular vein and the right subclavian vein and attached to the circuit outlet [16]. The left femoral artery was cannulated with a 24-gauge catheter (Insyte™ 0.7 × 19 mm, BD) to monitor systemic arterial pressure and to collect arterial blood samples for arterial blood gases, coagulation parameters and complete blood cell analyses [17]. The left femoral vein was cannulated with a 22-gauge catheter (Insyte™ 0.9 × 25 mm, BD) to administer methylprednisolone sodium succinate and sodium bicarbonate [17].

### ECMO circuit

The ECMO circuit is depicted in Fig. 1a. The circuit consisted of drainage and return tubing, a roller pump (505U Digital Peristaltic Pump, Watson Marlow, Wilmington, MA) and a custom-designed mock-oxygenator. The mock-oxygenator is a small, 3D-printed flow cell designed to simulate the local geometry and blood flow patterns of a commercially available membrane oxygenator hollow fiber bundle. However, the oxygenator is incapable of transferring gases because it is constructed from solid plastic. Therefore, this device is called a “mock-oxygenator.” While unable to transfer gas, its low prime volume, blood flow patterns, and surface area to blood volume ratio make it ideal for evaluating thrombus formation.

The mock-oxygenators were designed using Solidworks (Dassault Systemes SolidWorks Corp., Waltham, MA) to have a flow chamber that is 7.3 mm wide by 3 mm tall by 20 mm long, with a barbed inlet and outlet for 1/16 inch inner diameter (ID) tubing (Fig. 1b). To represent the hollow fibers of the fiber bundle, 345 rods with a 380 μm diameter were spaced evenly inside the mock-oxygenator to create a packing density of 50% (Fig. 1b). The chamber was then manufactured using a DLP™ 3D printer (Ember Autodesk, San Rafael, CA) for ease of repeatability and uniformity. A clear acrylate resin was used (PR-48, Colorado Polymer Solutions, Boulder, CO), which is most similar to urethane acrylate plastic. After printing, the flow chamber was rinsed with 60 mL of isopropyl alcohol from both the inlet and outlet to remove uncured resin. The mock-oxygenator was postcured with a UV lamp for 20 min and then sterilized using UV ozone for 15 min.

The mock-oxygenator was then placed in the ECMO circuit, as shown in Fig. 1a. T-connectors were used before and after the mock-oxygenator to monitor its inlet and outlet pressures and allow calculation of blood flow resistance. Heparin was administered through the inlet pressure line, and norepinephrine was administered through the outlet pressure line. Tygon tubing (E-3603, Fisher Scientific, Hampton, NH) with an ID of 1/16 inch was used to connect all circuit components. The total length of the circuit was 60 cm, and the total priming volume was 2.5 mL, of which the miniature mock-oxygenator constituted 0.3 mL with a surface area of 0.0015 m^2^.

### Experimental protocol

**Fig. 2:**
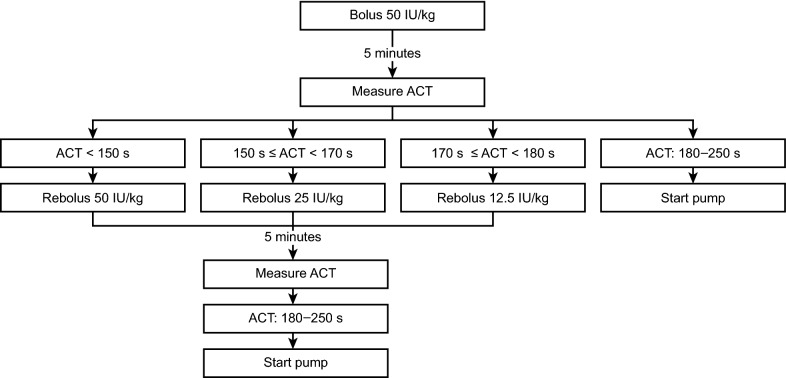
Protocol of dosage of heparin. Extracorporeal membrane oxygenation was started after the activated clotting time (ACT) level reached 180–250 s. First, 50 IU/kg heparin was bolused, and the ACT level was measured after 5 min. If the ACT level was less than 150 s, another 50 IU/kg heparin was rebolused. If the ACT level was between 150 and 170 s, another 25 IU/kg heparin was rebolused. If the ACT level was between 170 and 180 s, another 12.5 IU/kg heparin was rebolused

The circuit was flushed with 2 L/min carbon dioxide for 10 min and then primed with normal saline. Rats were randomly assigned to either the low-dose (LD) heparin group (n = 3) or the high-dose (HD) heparin group (n = 3) and placed on A-V ECMO at a blood flow rate of 1.9 mL/min. In both groups, 50 IU/kg heparin was bolused to achieve an activated clotting time (ACT) level of 180–250 s before initiating ECMO. An ACT level was measured five minutes after the bolus, and additional heparin boluses were given to maintain ACT between 180 and 250 s (Fig. 2). The heparin dose was determined based on previous studies [18, 19]. After flow through the circuit was started, heparin was administered at 15 IU/kg/h in the LD group throughout the experiment. In the HD group, heparin was administered at 50 IU/kg/h for one hour after initiating flow and then reduced to 25 IU/kg/h and maintained throughout the experiment. The doses of continuous administration of heparin were based on the results of previous studies [20] and preliminary experiments performed in our laboratory.

During the experiment, mean arterial pressure (MAP), heart rate (HR) and peripheral capillary oxygen saturation (SpO_2_) were monitored continuously and recorded every 15 min. The MAP was monitored using a Transpac IV pressure transducer (Transpac IV, icumedical, San Clemente, CA) connected to a BIOPAC MP150A-CE data acquisition system (BIOPAC Systems, Inc., Santa Barbara, CA). The HR and SpO_2_ were monitored by a Heska VetOx Plus 4800 Vital Signs Monitor (Heska, Loveland, CO). MAP was maintained above 60 mmHg by administering norepinephrine (0–0.5 mcg/kg/min). A bolus of 50 mg/kg methylprednisolone sodium succinate was also administered at the initiation of flow, and sodium bicarbonate was administered when the bicarbonate concentration (HCO_3_^−^) level was lower than 20 mmol/L according to the calculation (dose of sodium bicarbonate (mmol) = 0.3 × base deficit (mmol/L) × weight (kg)).

Blood samples for arterial blood gas (ABG) parameters (partial pressure of arterial oxygen, PaO_2_; PaCO_2_; pH; HCO_3_^−^; and lactate), white blood cell count (WBC), platelet count (Plt), total hemoglobin (Hb), hematocrit (Hct), and ACT were obtained from blood samples acquired through the femoral artery catheter. The ABG parameters and ACT were measured prior to starting ECMO and at one, four and eight hours following initiation of ECMO. The hematological parameters and bleeding time were measured prior to ECMO and one and eight hours following initiation. The ABG parameters were analyzed using a BL800 FLEX blood gas analyzer (Radiometer, Copenhagen, Denmark). Hematological parameters were measured with a hematology analyzer (Genesis™, Oxford Science, Oxford, CT). The WBC and Plt were then corrected for hemodilution during circuit priming using the formula corrected data = raw data × Hct (baseline)/Hct (sample time). The ACT was measured using a Hemochron Signature Elite (Accriva Diagnostics, Piscataway, NJ). The bleeding time was measured by inserting a 23-gauge needle 1 cm into the tail vein 1 cm from the tip of the tail. The needle was withdrawn immediately, and blood was blotted every five seconds with filter paper (Whatman™, Fisher Scientific) until bleeding stopped. The time at which no blood could be blotted on the filter paper was defined as the bleeding time. Mock-oxygenator inlet and outlet pressures were monitored using a BIOPAC data acquisition system (BIOPAC Systems, Inc.) in the same fashion as MAP monitoring and used to calculate resistance by dividing the inlet outlet pressure difference by the flow rate.

The experiment continued for 8 h or until the mock-oxygenator failed. The mock-oxygenator was considered to have failed when its blood flow resistance reached three times its baseline resistance. After the experiment was complete and the circuit was disconnected from the animal, the final thrombus weight was also measured. The mock-oxygenator was weighed before the experiment and immediately afterwards, and the change in mock-oxygenator weight was recorded as the thrombus weight.

### Statistical analysis

Descriptive variables are presented as the means ± standard error. Intergroup comparisons for weight and thrombus weight were assessed for significance using Student’s *t*-test. A linear mixed model was used to examine significant differences between heparin dose groups using the data taken during ECMO application (t > 0). Heparin dose, time, and the interaction of heparin dose and time were used as fixed effects, and the animal ID was used as a random effect. To specifically examine changes in variables from baseline (t ≤ 0) over time, additional linear mixed models were run for each specific heparin group, using time as the sole fixed effect and the animal ID as a random effect. All statistical analyses were performed with STATA software (version 13.0; Stata Corp LP, College Station, TX). All statistical tests were two-sided, and a p-value < 0.05 was considered significant.

## Results

ECMO was successfully established in three rats from each group. Rats weighed 507 ± 48 g in the LD group and 590 ± 21 g in the HD group (p = 0.19).

### Coagulation

#### Level of anticoagulation

**Fig. 3:**
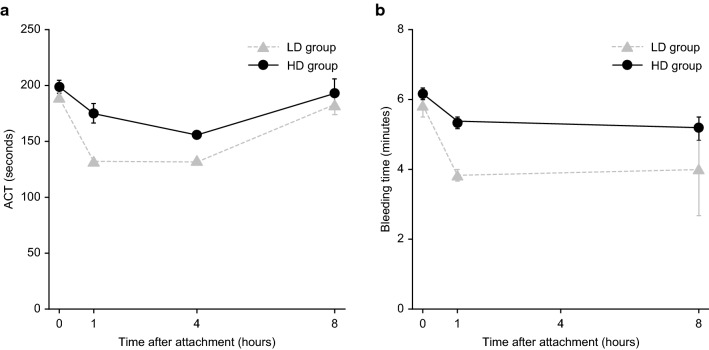
ACT level and bleeding time throughout the study. The ACT level in the HD group was higher than that in the LD group (**a**). In accordance with this, the bleeding time in the HD group was longer than that in the LD group (**b**). Error bars represent standard error of the mean. *ACT* activated clotting time, *LD* low-dose heparin, *HD* high-dose heparin

The mean ACT values before initiating ECMO were 189 ± 3 in the LD group and 198 ± 6 in the HD group (p = 0.25). One rat in the LD group needed an additional 25 IU/kg bolus of heparin, and one rat in the HD group needed an additional 100 IU/kg bolus to reach the target ACT range (180–250 s). During ECMO, the ACT for the HD group ranged from 152 to 220 s, which was similar to the clinical target range. In contrast, the ACT for the LD group during ECMO ranged from 120 to 200 s. The ACT in the HD group was significantly higher than that in the LD group (p < 0.05) (Fig. 3a). Accordingly, the bleeding time in the HD group was consistently higher than that in the LD group, but this difference only approached and did not reach statistical significance (p = 0.06) (Fig. 3b).

**Fig. 4:**
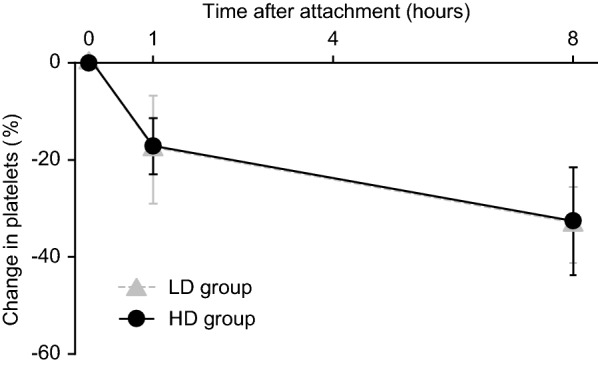
Change in platelet count from baseline measurement. The platelet count decreased throughout the experiment but showed no significant difference between the two groups. Error bars represent standard error of the mean. *LD* low-dose heparin, *HD* high-dose heparin

The platelet count decreased significantly throughout the experiment (p < 0.05) in both groups but showed no significant difference between the two groups (p = 0.94). On average, the platelet count decreased by 33 ± 8% in the LD group and 33 ± 11% in the HD group from baseline after eight hours of ECMO (Fig. 4).

#### Thrombus formation

**Fig. 5:**
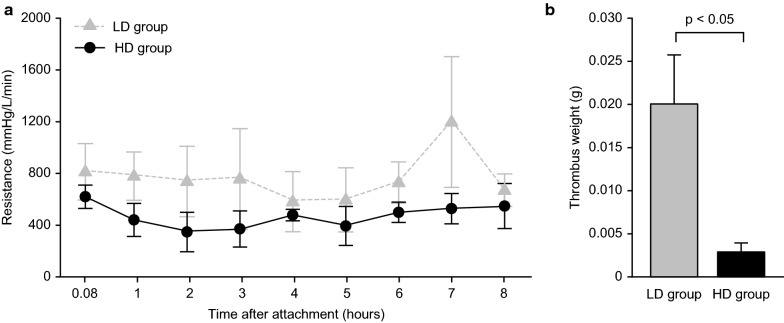
Resistance and thrombus weight of the mock-oxygenator. The resistance of the mock-oxygenator in the LD group was higher than that in the HD group throughout the study (**a**). The LD group showed a significantly higher thrombus weight than the HD group (**b**). Error bars represent standard error of the mean. *LD* low-dose heparin, *HD* high-dose heparin

The LD group mock-oxygenators had consistently higher resistance than those in the HD group (Fig. 5a) for the first seven hours of the study. By seven hours, the resistance in the LD group had increased to 1200 ± 503 vs 533 ± 117 in the HD group. However, this difference suddenly became negligible at eight hours, potentially due to thrombus detachment in the LD group. This change and within group variability in thrombus formation led to there being no significant differences between the two groups (p = 0.72).

Figure 5b shows the thrombus weight measured from the deposited thrombus inside the mock-oxygenators detached after each experiment. The LD group showed a significantly higher thrombus weight than the HD group (0.02 ± 0.006 g and 0.003 ± 0.001 g, respectively (p < 0.05)).

### Physiology

#### Hemodynamics

**Fig. 6:**
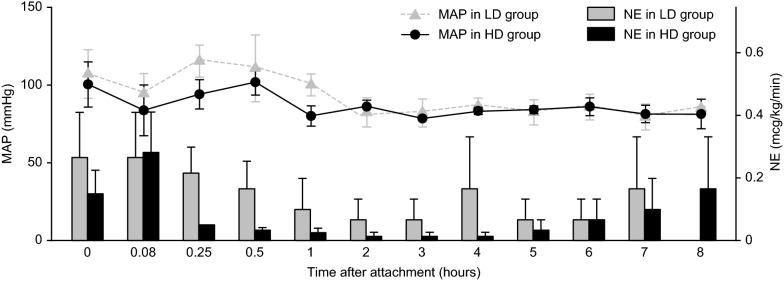
The trend of MAP and the amount of NE. The MAP decreased 5 min after initiating ECMO but remained stable one hour after initiating ECMO in both groups. In response to this, additional norepinephrine was required at the beginning in both groups. Error bars represent standard error of the mean. *LD* low-dose heparin, *HD* high-dose heparin, *MAP* mean arterial pressure, *NE* norepinephrine

Figure 6 shows the hemodynamic data during ECMO. Five minutes after the start of ECMO, MAP temporarily decreased by 13 ± 14 mmHg in the LD group and by 17 ± 15 mmHg in the HD group but stabilized one hour after the start. Norepinephrine infusion rates of 0.27 ± 0.15 and 0.28 ± 0.13 mcg/kg/min, respectively, were required in the LD and HD groups five minutes after the start of ECMO. The infusion rates decreased one hour after ECMO, reached their lowest values from two to four hours, and increased at the end of the experiment. There were no significant differences between the two groups (p = 0.88).

#### Hematocrit, hemoglobin, and white blood cell counts

**Fig. 7:**
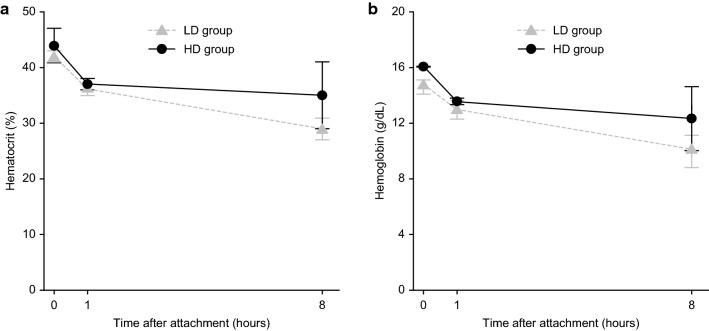
Change in hematocrit and concentration from baseline measurement. Hematocrit and hemoglobin concentrations were decreased throughout the study. Error bars represent standard error of the mean. *LD* low-dose heparin, *HD* high-dose heparin

Hct and Hb decreased gradually but significantly (p < 0.05) over time in both groups due to hemodilution caused by circuit priming, blood collection, crystalloid infusion, and inability to transfuse (Fig. 7). The Hct decreased from 42 ± 1% to 36 ± 1% at 1 h after ECMO initiation and 29 ± 2% at eight hours after ECMO initiation in the LD group. Similarly, the Hct decreased from 44 ± 3 to 37 ± 1% at 1 h and 35 ± 6% at 8 h in the HD group. The Hb level decreased from 14.6 ± 0.5 to 12.8 ± 0.5 g/dL at 1 h and 10.0 ± 1.2 g/dL at 8 h in the LD group. The Hb level decreased from 16.1 ± 0.0 to 13.6 ± 0.2 g/dL at 1 h and 12.3 ± 2.3 g/dL at 8 h in the HD group. However, there was no difference between the two groups in terms of Hct (p = 0.71) and Hb (p = 0.631) levels.

The WBC count was increased from 8.4 ± 1.8 K/µL to 11.8 ± 4.8 K/µL at 1 h and 11.0 ± 5.3 K/µL at 8 h after ECMO in the LD group. Similarly, the WBC was increased from 9.5 ± 2.2 to 11.7 ± 1.4 K/µL at 1 h and 12.3 ± 2.5 K/µL at eight hours after ECMO in the HD group. However, this increase was not significant (p = 0.50 for the LD group and p = 0.25 for the HD group) in either group, and there was no significant difference between the two groups throughout the experiment (p = 0.97).

#### Arterial blood gas

The changes in arterial blood gas values during the experiments are presented in Table 1. The pH decreased slightly over time in both groups (p = 0.11 for the LD group and p = 0.18 for the HD group). The PaO_2_ was stable in both groups during ECMO. The PaCO_2_ increased significantly (p < 0.05) over time in the LD group. The reason was that the respiratory rate was set below 50 breaths per minute because PaCO_2_ at one hour after attachment was lower than 35 mmHg in one rat. The PaCO_2_ in the HD group was stable (p = 0.35). The HCO_3_^−^ in the LD group changed significantly because bicarbonate was administered according to the HCO_3_^−^ levels (p < 0.05). The HCO_3_^−^ levels in the HD group were stable (p = 0.47). One rat in the LD group presented with a high lactate level eight hours after initiating ECMO, although all other rats had lactate levels below 20 mg/dL.

**Table 1:** Data from blood gas analysis throughout the study

Variables	Groups	Time after attachment (hours)			
		0	1	4	8
pH	LD	7.38 ± 0.04	7.31 ± 0.03	7.32 ± 0.04	7.32 ± 0.06
	HD	7.40 ± 0.07	7.38 ± 0.10	7.33 ± 0.07	7.27 ± 0.06
PaO_2_ (mmHg)	LD	369 ± 12	324 ± 40	333 ± 10	351 ± 13
	HD	341 ± 13	317 ± 7	350 ± 23	370 ± 8
PaCO_2_ (mmHg)	LD	36.3 ± 4.6	33.5 ± 1.3	39.6 ± 0.8	50.1 ± 7.3
	HD	33.2 ± 11.7	37.2 ± 5.3	40.4 ± 10.0	37 ± 3.5
HCO_3_^−^ (mmol/L)	LD	20.8 ± 1.0	16.3 ± 1.2	19.9 ± 1.4	24.5 ± 0.4
	HD	18.4 ± 3.2	21.2 ± 1.9	19.5 ± 1.1	20.8 ± 1.0
Lactate (mg/dl)	LD	7 ± 1	9 ± 2	10 ± 3	31 ± 22
	HD	7 ± 2	10 ± 1	14 ± 1	17 ± 1

Data are expressed as mean ± standard error

*LD* low-dose heparin, *HD* high-dose heparin

## Discussion

An eight-hour ECMO model was successfully established in rats and can be used to examine differences in circuit thrombus formation using different anticoagulation methods. The model is simple, economical, safe, and reproducible. Unlike a large animal model, a single operator can perform experiments with this model independently, including multiple experiments at the same time. In addition, this model could be produced in the same way in different laboratories using standardized rat strains, allowing better comparison between different laboratories.

To our knowledge, there is no previous rat model designed for investigating ECMO coagulopathy. Several studies have reported the establishment of an animal model of cardiopulmonary bypass (CPB) and venoarterial (V-A) ECMO in rats [21–35] (Table 2). However, these models were developed to investigate the pathophysiology [21, 25, 28–32, 34], complications [23, 24, 26, 27, 30, 32], and effectiveness of methods of cardiac arrest [33] specific to CPB and V-A ECMO, or to evaluate membrane oxygenators [22, 35]. CPB is usually performed by central cannulation through median sternotomy because its purpose is complete replacement of cardiopulmonary function during short-term cardiac surgery. The CPB circuit requires an open reservoir, which requires anticoagulation therapy with high-dose heparin. In contrast, ECMO provides long-term cardiac or pulmonary support for severe cardiac or respiratory failure in the intensive care unit by peripheral cannulation. The ECMO circuit is a closed system without a reservoir. During ECMO, bleeding and thrombus formation are managed by titration of minimal anticoagulation. Therefore, previous rat models have certain limitations for investigating ECMO coagulopathy. First, they require a venous reservoir and large surface area of the oxygenator, which strongly promote thrombus formation. Second, the dosage of heparin used in most previous models is 500 IU/kg, which greatly exceeds that used in ECMO (Table 2). Even in the V-A ECMO model, a venous reservoir and high doses of heparin were used [33, 35]. Third, they require median sternotomy or a custom-designed cannula and application of transesophageal echocardiography (TEE) [23] to avoid drainage failure. Finally, all of them depend on a large priming volume of between 8 and 40 mL, which represents approximately 25 to 200% of the circulating blood volume of a 250 to 550 g male Sprague–Dawley rat (blood volume, 64 mL/kg). This problem is compounded by the need to take blood samples during support for the assessment of coagulation and other physiological variables. The combination of a large circuit volume plus blood sampling can quickly result in excessive hemodilution. As a result, some of the models require donor blood to prime the circuit [21, 23, 24, 27]. These disadvantages limit the use of these models for coagulation research in ECMO.

**Table 2:** Previously published rat cardiopulmonary bypass and venoarterial ECMO models

	Weight (grams)	Oxygenator	Surface area of oxygenator (m^2^)	Priming volume of circuit (oxygenator) (mL)	Priming solution	Duration of extracorporeal circulation (hours)	Dosage of heparin
Popovic et al. [21]	250–300	Homemade	0.048–0.064	13.7 (11.3)	blood	3	N/A
Alexander and Al Ani [22]	250	Homemade	0.017	12 (4.4)	crystalloid	6	1 mg/kg
Grocott et al. [23]	350–400	Homemade	0.33	40 (27)	blood	1	150 IU
Fabre et al. [24]	475–550	Homemade	N/A	35 (15)	blood	2	500 IU/kg
Gourlay et al. [25]	350–450	Homemade	0.05	12 (4)	colloid	1	1000 IU/kg
Hamamoto et al. [26]	350–420	HPO-003	0.03	9 (3.3)	colloid	1	200 IU/kg
Dong et al. [27]	450–550	Micro-1	0.05	16 (3.5)	blood	1	500 IU/kg
You et al. [28]	430–475	Homemade	0.02	9.5 (2.8)	colloid	1	500 IU/kg
Modine et al. [29]	422.9 ± 32	Homemade	0.05	10 (4)	colloid	1	500 IU/kg
Gunzinger et al. [30]	400–500	Homemade	0.063	8 (4)	colloid	1	500 IU/kg
Cresce et al. [31]	250–350	Homemade	0.045	30 (6.3)	crystalloid	2	500 IU/kg
Zhang et al. [32]	250–300	Micro-1	0.05	< 12 (3.5)	colloid	0	500 IU/kg
Ali et al. [33]	250–350	Homemade	N/A	8 (N/A)	crystalloid	1	100 IU
Chang et al. [34]	450–550	Micro-1	0.05	20 (3.5)	crystalloid	1	500 IU
Edinger et al. [35]	350–400	Micro-1	0.05	9 (3.5)	colloid	2	250 IU
	350–400	SAMO	0.05	11 (7)	colloid	2	250 IU
Present study	450–550	3D-printed	0.0015	2.5 (0.3)	crystalloid	8	15–50 IU/kg/h

Data are expressed as mean ± standard deviation [29]

*N/A* not available, *IU* international unit

These problems were addressed in the rat ECMO model performed in this study. First, unnecessary aspects of CPB that are not present in ECMO were eliminated, including the venous reservoir and median sternotomy. Second, a pumped A-V configuration was chosen instead of the more common venovenous (V-V) or V-A configurations to avoid complications that occur when the cannula tip is not perfectly placed near the right atrium. This requires TEE, a custom-designed cannula and gravity blood drainage to avoid inserting the cannula too little, resulting in poor venous drainage, or too far, resulting in right ventricular damage and/or arrythmias. Furthermore, we decided against pumpless A-V support to avoid changes in circuit flow that occur due to arterial blood pressure changes under anesthesia. Third, the oxygenator was manufactured using 3D-printing, which allows a smaller prime volume and greater reproducibility. The commercially available Micro-1 (Kewei Medical Instrument Inc., Dongguan, China) has a surface area of 0.05 m^2^ and requires a 3.5 mL priming volume [27, 32, 34, 35]. Similarly, commercially available HPO-003 (Senko Medical Co., Ltd, Tokyo, Japan) has a surface area of 0.03 m^2^ and a 3.3 mL priming volume [26]. Furthermore, gas flows countercurrent to blood flow in both oxygenators and blood flows inside fibers in HPO-003 oxygenator. Thus, the ratio of surface area to blood volume in both oxygenators is larger than that in clinical oxygenators (0.005 m^2^/mL), and the blood flow patterns are not the same as modern oxygenators that feature external, cross-flow of blood. Other custom-built oxygenators used in these studies required a priming volume between 2.8 and 27 mL [21–25, 28–31, 33]. Unlike these oxygenators, the 3D-printed microfluidic device in this study has a 0.3 mL priming volume and a surface area of 0.0015 m^2^.

As a result of all these changes, the total priming volume of the circuit was reduced to 2.5 mL. This enabled a small animal model with hemodynamic stability for eight hours and reduced hemodilution, which is crucial for investigating coagulopathy during ECMO. In previous CPB animal models, the MAP fell to 44–66 mmHg during CPB [23–25, 28, 29, 34]. The mean MAP decreased from 121.1 mmHg to 41 mmHg at 30 min after the initiation of CPB using the Micro-1 without blood transfusion [34]. In this study, MAP decreased when ECMO was initiated but was maintained at approximately 80 mmHg thereafter. The reason for hypotension at the beginning of ECMO was due to blood being pulled from the drainage cannula and hemodilution with the circuit prime volume. The need for more vasopressors at the end of the experiment was likely due to progressive vasodilation due to prolonged anesthesia and repeated blood sampling. Lactate levels were normal throughout the experiment in all rats except one, and thus, adequate tissue perfusion and oxygenation were maintained.

Hemodilution was also minimal. Previous studies have reported that Hct and Hb concentrations were decreased by 35–50% from baseline in CPB without donor blood due to hemodilution from priming and laboratory sampling [25, 28, 30, 32, 34]. The Hb level at two hours after the extracorporeal circulation using SAMO (M. Humbs, Valley, Germany) and Micro-1 was 7.1 g/dL and 6.9 g/dL, respectively, even though colloid fluids were used for priming [35]. However, Hct and Hb concentrations were decreased by only 20–30% from baseline at eight hours after the extracorporeal circulation in this current model without donor blood or colloid fluids. The final values of Hct and Hb were thus approximately 30% and 10 g/dL, respectively, which more closely matches clinical ECMO.

Collectively, rat CPB and ECMO models with commercially available oxygenators require large priming volumes, causing hemodilution and hypotension or requiring transfusion or colloid fluids. These disadvantages are unacceptable for thrombus research. The rat ECMO model with the 3D-printed mock-oxygenator has the following additional advantages: (1) the mock-oxygenator maintains the geometry and thus the blood flow patterns and surface area to blood volume ratio of a clinical oxygenator; (2) the mock-oxygenator can be fabricated quickly and cost-effectively; (3) cannulation can be easily established without a surgeon; (4) the use of pumped A-V eliminates poor blood flows due to occlusion of the drainage cannula by the low pressure, flexible vein during V-V or V-A ECMO while allowing simple control of blood flow rates unlike A-V; and (5) the system has the smallest priming volume available, which enables long-term experimentation under anesthesia without requiring transfusion or colloid fluids. For all these reasons, this system allows a more accurate assessment of circuit thrombus formation than systems using commercially available miniature oxygenators.

This model would, therefore, be effect at investigating solutions to cannula and oxygenator thrombosis, including those reported in COVID-19 patients [36], and bleeding propensity during ECMO [3, 37, 38]. This model is similarly useful to investigate the optimal extent of anticoagulation (e.g., optimal activated partial thromboplastin time or ACT), the effectiveness of new anticoagulants such as oral FXa agents or specific FXII [10] or FXI inhibitors, and biomaterial surface coatings [8] to minimize thrombus, all of which are urgently needed, particularly during the COVID-19 pandemic [36]. However, our model does not evaluate the effect of anticoagulation strategies on the deterioration of the oxygenator’s gas exchange due to thrombus formation.

Most importantly, our results provide a guide to heparinization methods in the rat model. Most of the previous CPB and V-A ECMO models did not measure clotting times throughout the study to evaluate the anticoagulation level [21–26, 28–33]. Other experimental studies have maintained high ACT levels of over 300 s [27, 34]. However, these are different from the clinical practice of ECMO and are not suitable for studying anticoagulation therapy in the setting of clinical ECMO. To our knowledge, there are no previous studies that showed the relationship between ACT level and heparin dosage in a rat model. In this study, ACT was controlled within the range of 152 to 210 s in the HD group. The majority of institutions utilize an ACT between 140 and 220 s [5]. Therefore, this same dose of heparin should serve as a control group for future studies examining new anticoagulation regimens, including surface coatings and systemic anticoagulants. In addition, this study demonstrated lower thrombus weight and longer bleeding times with an increasing level of heparin anticoagulation. In a meta-analysis by Sy et al. [5] patients with a higher ACT target (> 180 s) bled more than patients with lower targets (< 180 s). However, a greater level of anticoagulation was associated with lower thromboembolic events [5]. An increase in blood flow resistance is one of the commonly used criteria for oxygenator exchange. However, it remains a vague indicator and reliable only in advanced thrombus stages [39, 40]. Thus, only 5–20% of oxygenators that require an exchange show increases in blood flow resistance [39, 41]. Our results are consistent with clinical studies and suggest that mock-oxygenators could be useful in investigating thrombus formation.

In contrast, the platelet count decreased over time in both groups, with no significant difference between groups (p = 0.94). Thrombocytopenia during ECMO has been well documented during in vivo and clinical ECMO studies [8, 22, 42]. This observation is typically attributed to platelets adhering to the circuit and oxygenator surfaces [43] and damage or activation by the pump [44]. However, emerging studies suggest that this effect may not be due to gross thrombus formation in the ECMO circuit [8, 10]. This rat ECMO study is thus consistent with these studies’ suggestion that the degree of thrombocytopenia does not appear to be related to the degree of thrombus formation.

Despite the positive results, this study has several potential limitations. First, circuit inflammation was evaluated in this study only by white blood cell count. To understand this aspect of blood biocompatibility, a more complete study is needed to examine complement activation and cytokine generation. Second, our model does not use the polymethylpentene (PMP) hollow fibers that form clinical ECMO oxygenator gas exchange membranes. The simulated fibers in our devices are solid urethane acrylate (UA) posts, and thus, one cannot study gas exchange. Urethane acrylate and PMP are both hydrophobic polymers, and thus, the thrombusting process is similar. However, the rate of thrombus formation may be slightly different due to the different chemical structures. Last, rats have a platelet count three to six times higher than that of humans, and thus, the rate of thrombus formation and thrombus composition may be different, even at the same ACT [45].

## Conclusions

Ultimately, this rat ECMO model using a 3D-printed oxygenator demonstrates significant, measurable changes in thrombus formation based on the mode of anticoagulation and is thus an effective model for investigating ECMO-related coagulopathy. This model could be applied to further studies of ECMO-associated thrombus formation, including disease-specific coagulopathies (e.g., sepsis and COVID-19) and new anticoagulation strategies.

## Data Availability

The datasets used and/or analyzed during the current study are available from the corresponding author on reasonable request.

## Abbreviations

ECMO: Extracorporeal membrane oxygenation
A-V: Arteriovenous
PaCO_2_: Partial pressure of arterial carbon dioxide
ID: Inner diameter
LD: Low-dose
HD: High-dose
ACT: Activated clotting time
MAP: Mean arterial pressure
HR: Heart rate
SpO_2_: Peripheral capillary oxygen saturation
ABG: Arterial blood gas
PaO_2_: Partial pressure of arterial oxygen
HCO_3_^−^: Bicarbonate concentration
WBC: White blood cell count
Plt: Platelet count
Hb: Hemoglobin
Hct: Hematocrit
CPB: Cardiopulmonary bypass
V-A: Venoarterial
TEE: Transesophageal echocardiography
N/A: Not available
IU: International unit
V-V: Venovenous
PMP: Polymethylpentene
UA: Urethane acrylate
